# Serum tissue plasminogen activator after cycling with blood flow restriction

**DOI:** 10.1530/VB-24-0008

**Published:** 2025-02-17

**Authors:** Josh B Landers, Melissa Allen, Ibrahim Oladele, Leah Lowe, Nawab Ali, Jacquie Rainey, James Fletcher, Korben Landers

**Affiliations:** ^1^ Lyon College Institute of Health Sciences, Lyon College of Dental Medicine, Little Rock, Arkansas, USA; ^2^ UCA Department of Physical Therapy, Conway, Arkansas, USA; ^3^ Center for Integrative Nanotechnology Sciences, University of Arkansas at Little Rock, Little Rock, Arkansas, USA; ^4^ Department of Biology, University of Arkansas at Little Rock, Little Rock, Arkansas, USA; ^5^ Department of Health Sciences, Conway, Arkansas, USA; ^6^ Psychology Department, University of Arkansas at Little Rock, Little Rock, Arkansas, USA

**Keywords:** blood flow restriction exercise, tissue plasminogen activator, vascular occlusion training

## Abstract

Blood flow restriction exercise (BFRE) is a therapeutic approach traditionally used to facilitate muscular strength and hypertrophy. Emerging evidence has identified its benefits on other systems and metabolic processes. The emphasis of this study was to examine potential impact of BFRE on serum levels of tissue plasminogen activator (tPA). Eighteen healthy adults (nine males, nine females; mean age: 34.44 ± 9.97) were randomized into groups to perform cycling either with or without blood flow restriction (BFR). Blood samples were collected before and after exercise to analyze serum concentrations of tPA. Significance in tPA between exercise groups did not reach significance but did show a large effect size (0.14) in favor of the BFR group. The trend suggests that this study was underpowered to reach significance. Further research should continue examining the impact of BFRE on serum levels of tPA. This methodology could be adapted to other populations to increase generalizability of results.

## Introduction

Blood flow restriction exercise (BFRE), also known as vascular occlusion training, is a therapeutic approach used to facilitate muscular strength and hypertrophy in individuals who are unable to tolerate heavy loads (1). While foundational studies have examined the utility of BFRE in rehabilitation of orthopedic injuries and general deconditioning, recent evidence supports novel utilization of BFRE to alter serum tissue plasminogen activator (tPA) concentration.

Previous literature examining BFRE have found very few cases of deep vein thrombosis (DVT) reported (0.06%) in over 30,000 BFRE training sessions (2). In fact, the incidence reported in these reviews is actually less than that of the general Asian population (∼0.2–0.26%) (3). These findings galvanized Nakajima and coworkers to investigate how BFRE may alter serum levels of tPA. In the first of the two study protocols, a group comprised of six healthy adult males (mean age 48 ± 5 years; mean height, 1.71 ± 0.2 meters; mean weight, 70.1 ± 4.3 kg) and had the blood flow restriction (BFR) cuffs inflated on the upper thighs to 160 mmHg pressure without exercise for fifteen minutes. At that time, the cuffs were deflated and blood was drawn followed by resting in a seated position for fifteen minutes. Finally, the cuffs were reinflated, and they performed the following exercises: i) toe flexion and extension (20 reps, 2 sets), ii) ankle dorsiflexion (20 reps, 2 sets), iii) ankle plantar flexion (20 reps, 2 sets), iv) unilateral knee extension (20 reps each) and v) unilateral leg press motion (20 reps each). Economy class syndrome is a serious problem in airflight, where the activation of the coagulation cascade and subsequently thrombus formation may occur. Protocol one aimed to determine the effects of the BFRE on the hemostasis under airflight conditions by conducting the study in a hyperbaric chamber simulating 8000 feet altitude. tPA activity increased in the hyperbaric chamber from 0.26 ± 0.016 to 0.32 ± 0.04 U/mL, with the BFR cuffs inflated (*n* = 6, *P* < 0.05), but a greater increase was noted when exercises were combined with BFR (0.34 ± 0.03 U/mL, *n* = 6). Prothrombin time, thrombin time, fibrinogen, factor 8 and platelet counts did not change with BFR only and BFRE.

In protocol two, seven men (mean age: 31.6 ± 1.1 years; mean height: 1.76 ± 1.6 meters; mean weight: 75.3 ± 3.9 kg) performed four sets (one set of 30 repetitions followed by three sets of 15 repetitions) of leg press exercises at an intensity of 30% 1-RM (repetition maximum) with and without BFR after 24 h of bed rest, which is also known to increase the risk for coagulation. The researchers found that tPA antigen did not change significantly during the leg press exercises without BFR (2.2 ± 0.1 g/mL at rest and 2.3 ± 0.1 ng/mL immediately after the exercise), but did significantly increase from 2.1 ± 0.1 to 2.7 ± 0.2 ng/mL (*n* = 7, *P* < 0.05) when exercises were combined with BFR. In addition, the BFRE group had no significant change in prothrombin time, fibrinogen, factor 10 or platelet count.

In both protocols, BFRE did not induce fibrin formation as assessed by fibrin D-dimer, fibrin degradation products (FDP) or plasminogen activator inhibitor (PAI)-1. Rather, the study concluded, in healthy men, BFR alone or BFRE induced potentially favorable changes in fibrinolytic factors (4). A potentially significant limitation of this study is that a within-subjects methodology for repeated measures was used, with participants only having a 15-min washout time between conditions. This short washout period could have confounded the increase observed in tPA, particularly after the rest plus KAATSU pressure condition. The study participants, however, were described as being ‘untrained’, and other studies have found that ‘untrained’ participants have a diminished fibrinolytic response (5). This could indicate that the results were due to the intervention and not solely reliant on natural processes. Further research is needed to better understand these adaptations.

A randomized-controlled trial performed by Clark *et al*. (6) evaluated the acute effect of BFRE on markers of coagulation (fibrinogen and D-dimer), fibrinolysis and inflammation. tPA and high-sensitivity C-reactive protein (hsCRP) were obtained in response to exercise bouts separated by 4 weeks. Sixteen individuals (14 men, two women) between 18 and 30 years of age participated in the study. Nine participants performed BFRE training at an intensity of 30% RM strength with a tourniquet compression of 1.3 times their resting brachial systolic blood pressure (SBP) located on the proximal thighs, while seven participants performed high-load resistance exercise (HLE) at an intensity of 80% 1-RM strength without BFR. The BFRE protocol consisted of a knee extension exercise (isotonic, 2-s concentric and 2-s eccentric action, three sets with 90-s of rest between sets). The device maintained a constant pressure throughout the entire exercise protocol. Participants exercised to volitional fatigue.

A 30–40% increase in tPA antigen was shown after both BFRE and HLE (*P* = 0.01); however, no changes were observed in D-dimer, fibrinogen or hsCRP (*P* > 0.05). Both protocols did result in an increase in fibrinolytic activity without an associated change in coagulation or inflammation responses. The authors of the study noted that observations from their study should be restricted to the respective-dependent variables. For example, different conclusions may have been drawn if other markers of clotting such as PAI-1 had been observed. In addition, as with most of the BFRE studies, it is likely that the prescribed cuff pressure likely did not uniformly restrict blood flow similarly across all participants secondary to the disassociation between tourniquet pressure, underlying soft-tissue pressure and limb circumference. In addition, the training status of the subject, which has been shown to impact fibrinolytic response, was not reported (6). The training status is pertinent, in that trained participants present a higher tPA release when compared with untrained participants (5).

In 2019, Nascimento and coworkers performed a systematic review of the aforementioned studies investigating the effects of BFRE on hemostasis. A total of 127 participants were included across nine studies. One study included participants with ischemic heart disease, while eight studies included young and apparently healthy older adults (age range between 23 and 72 years) (4, 6, 7, 8, 9, 10, 11, 12, 13). The quality of the articles were appraised using a modified version of Downs and Black checklist on items including external validity, internal validity, reporting and statistical power. Studies were classified as being excellent (26–28), good (20–25), fair (15–19) or poor (≤14) (14). The average score of reviewed studies was 11.22 (range 9–13) and the level of evidence was IIB (e.g., low-quality randomized-controlled studies). Contributing to the less than desirable average are factors such as small sample sizes, underrepresentation of females and inconsistency of the occlusion pressures and protocols. As most of the reviewed studies failed to report the occurrence and incidence of adverse effects after BFR interventions, the authors of the review concluded that it is difficult to definitively advocate the safety of BFR to those with pathology due to the weak methodological study designs in the current literature. However, despite these concerns, the authors noted that the effects of short-term BFRE data effectively support the safety of BFR implementation for participants described as young, middle-aged with stable ischemic heart disease or apparently healthy older participants. The authors also conclude that long-term exercise with BFR using low-load resistance exercise via elastic bands is a relatively safe method for older adults regarding blood hemostasis (15).

Based on this epidemiological research and safety profile, BFRE was applied to a patient diagnosed with an active DVT in the following case study (written by the present author and colleagues), recently published in the Journal of Orthopedic and Sports Physical Therapy Cases (16). After extensive literature reviews on the utility of BFRE into various patient populations and discovering the effects of BFRE on metabolic and cellular level biomarkers, the current author implemented this novel approach into clinical practice by applying BFRE to the uninvolved extremities of a patient experiencing knee pain due to a postsurgical DVT. This exploratory case study provided anecdotal support for the novel utility of BFRE as an adjunct to medical management of DVT and served as the catalyst for the primary aims of this dissertation work.

The ability of BFRE to stimulate the fibrinolytic system could have potential in facilitating the dissolution of a thrombus (4, 6). Furthermore, BFRE can be applied to the uninvolved limbs to decrease the risk for venous stasis in the involved extremity. The purpose of this case report was to investigate the incorporation of BFR in facilitating the dissolution of a DVT secondary to an increase in tPA levels in the blood. In this case study, the participant was a 40-year-old male (height, 5 feet 10 inches; weight, 197 pounds; body mass index, 28.3), who developed a DVT after having lateral meniscectomy. Medical management of the DVT began immediately after the positive ultrasound test 5 days after surgery. BFRE was added to physical therapy beginning approximately 90 days after surgery with the expectation of expediting the dissolution of the DVT due to increase in systemic tPA, as demonstrated in previous studies (4, 6). Physical therapy sessions included a ten minute warm-up consisting of BFR exercise on the Assault bike (intensity between 150 and 175 W and 45–50 r.p.m.), while wearing elastic, pneumatically controlled BFR bands (BStrong™, USA; 5 cm wide × 50 cm long) around the proximal region of both upper extremities (band pressure 160 mmHg) and the thigh of the uninvolved lower extremity at 300 mmHg. The cuff pressures were determined based on a previous report using BStrong™ elastic cuffs (17). After application and inflation of the elastic cuffs, Doppler ultrasound was performed by the physical therapist at the posterior tibial artery at the medial malleolus and the radial arteries at the lateral aspect of the wrist to ensure arterial inflow to the extremities receiving BFR. The Assault bike was used to combine BFR with exercise of bilateral upper and lower extremities.

At follow-up with his hematologist immediately after the 2.5-week period using BFRE, the venous duplex ultrasound showed no evidence of DVT. The results of this case could indicate that a BFR-induced increase in systemic tPA facilitated fibrinolysis, even though anticoagulation medication was concurrent with PT and BFR. No adverse side effects were reported. While it is possible that medication alone might have led to a resolution of the DVT, anticoagulants work to prevent clot formation and growth, but do not dissolve existing clots (18). Natural processes might also have facilitated the resolution of the DVT over time. Further studies are needed to address these confounds systematically. This case study could serve as a template and rationale for clinical trials to determine if BFR could be an efficacious adjunct in the clinical treatment of DVTs. BFR could promote fibrinolysis of a DVT if it increases systemic tPA, but more research is needed to establish a causal relationship. The authors noted that physical therapists should be highly selective and cautious when using BFR with an active DVT until further research substantiates its safety and efficacy. The aim of this study was to determine if tPA was significantly elevated in healthy, adult participants following a bout of BFR with low-load exercise of three extremities when compared to work-matched controls.

## Materials and methods

The study was approved by the University of Central Arkansas Institutional Review Board on May 4, 2023 (IRB no. 23-09). Eighteen healthy participants, aged 18–50 years (mean age 34.44 ± 9.97), were enrolled in the study. This sample size was derived from an *a priori* G*power™ calculations using a moderate-to-large effect size (*f* = 0.75) from a similar study using the same dependent variables to achieve a power of 0.85 (2, 3). An alpha value of 0.05 was predetermined for significance.

To obtain baseline data of a normative population, young, healthy adults with no history of previous chronic illness and those who have not had acute illness within the last three months were recruited to participate. Specifically, participants were excluded if they had a resting SBP exceeding 140 mmHg or a diastolic blood pressure exceeding 90 mmHg at the time of study participation, currently taking antihypertensive drugs, a body mass index ≥30, any orthopedic, an ankle brachial index between 0.90 and 1.26, neurological or cardiovascular diseases that would limit safe exercise, have a personal or family history of blood clotting disorder or reported smoking in the last six months as assessed by interviews and questionnaires before enrollment.

The participants were asked to fast for 12 h and avoid alcohol for 24 h before their scheduled data collection time to minimize variation in analytes and maintain consistency with other studies measuring tPA (4, 6). Data collection times were scheduled between 08:00 and 10:00 h for all participants to minimize diurnal variation in blood analytes.

Participants were randomly assigned to either the BFRE group or the work-matched control without BFR using the Excel random number generator. To ensure equal representation of males and females in both exercise groups of this study, a stratified block randomization of participants was utilized. For example, when a male participant was enrolled, he was first allocated to the male strata, and the group (BFRE or work-matched controls without BFR) was determined through block randomization applied to the male strata. The same assignment method was used for female participants. The blocks of four were computer-generated using an Excel random number generator.

Upon arrival, participants were instructed to rest quietly in a seated position for ten minutes to diminish postural effects on blood analytes. Following the allotted resting time, the investigator assessed each participant’s blood pressure to ensure it was within inclusion limits. If blood pressure was within inclusionary limits, the participant proceeded with blood sampling and exercise procedures. If blood pressure was outside inclusion limits, the participant rested in a seated position for an additional ten minutes and then blood pressure was reassessed at the end of that time. If the average of the two blood pressures did not meet inclusionary requirements, the participant was excluded from participation in the study. In addition, the ankle brachial index was used to assess peripheral vascular disease. The subject was positioned in a supine position, and brachial and ankle SBPs were obtained from both sides of the body using a Doppler probe. The SBP in the posterior tibial artery of the ankle was divided by the brachial SBP and expressed as an index. The normal range of ankle brachial index in young healthy adults is 1.02–1.26 (19). The cutoff for pathological indications of peripheral vascular disease is typically 0.90 (20). If the ankle brachial index fell outside this range, the participant was excluded.

Venous blood samples were obtained from the left antecubital vein immediately before and after exercise to assess potential changes in tPA levels in the blood following cycling exercise with or without BFRE. If the antecubital vein was problematic, the cephalic or basilic veins were used to obtain samples. Blood samples were collected in red top vials that promote blood coagulation for the determination of fibrinolytic variables. Pre-exercise, one vial of blood was obtained from a single blood draw. Upon sampling, vials were inverted to mix and placed on ice until processing via centrifuge. Blood samples were stored in vials coded with a unique, three-digit number to maintain participant confidentiality.

Following the initial blood draw, all participants began cycling on an Assault™ bike, which is a low-impact, high-output machine that combines the arm action of an elliptical trainer with the lower body and cardiovascular workout of a stationary bike. During the exercise, both lower extremities and the upper extremity not utilized for the blood draw were in motion. The participants were instructed to cycle at intensity between 150 and 175 W with 45–50 r.p.m. for 15 min. The target exercise duration and intensity utilized for this study mirrored those outlined in a previous BFR cycling study and the case report of the current researcher (16, 21). Ratings of perceived exertion (RPE) at a level of 11–12 (light exercise) on the Borg scale was targeted. This target RPE of 11–12 would present as minimal sweating and ability to talk easily (22). Throughout the exercise, the investigator engaged participants in conversation to assess their ability to talk without difficulty. If a participant was having difficulty talking (which describes RPE of 13–14), the intensity was reduced until they regained the ability to breathe easily during exercise, indicating that the desired RPE was re-established. In addition to monitoring RPE, each participant wore a finger heart rate monitor on the resting upper extremity. The investigator monitored each participant’s heart rate to ensure it was maintained within the desired range of 63% of their HR max plus or minus 10 beats per minute. This designated heart rate range corresponds to steady-state low-intensity exercise at approximately 40% VO_2_ max (23).

For participants in the BFRE group, the BFR cuff was applied to the arm opposite the blood draw, followed by the lower extremities. After BFR cuffs were applied, but before initiating exercise, Doppler ultrasound was performed on the posterior tibial arteries at the medial malleolus and the radial artery at the lateral aspect of the wrist to ensure arterial inflow to the extremities receiving BFR. During cycling, participants assigned to the BFRE group wore elastic, pneumatically controlled BFR cuffs (BStrong™, USA; 5 cm wide × 50 cm long) around the upper arm (above the elbow) on the arm opposite the blood draw (cuff pressure 160 mmHg) and around the upper thigh of both lower extremities (cuff pressure 300 mmHg). The cuff pressures were determined based on a previous report using BStrong™ elastic cuffs (17) and mirroring the current researcher’s recently published case study (16). Participants assigned to the work-matched control group performed the same cycling exercise on the Assault™ bike at previously described intensity without the BFR cuffs. For participants in both the BFRE and control group, the upper extremity from which the blood draw was obtained rested comfortably throughout the exercise.

### Blood serum analysis

Immediately following exercise, a second blood draw was taken from the same antecubital vein used for the initial draw. This additional vial of blood was used to extract serum for tPA analysis. Once the post-exercise blood draw was completed, the blood samples sat for 30 min to allow for coagulation. After 30 min, the vial was inverted, and if any movement occurred, the blood was allowed to sit for an additional ten minutes. This process was repeated until the blood was completely coagulated. At this point, it was processed via centrifugation at 2300–2800 r.p.m. for ten minutes, allowing serum and plasma to be separated. The serum was aliquoted off the blood cells using a pipette, transferred to an Eppendorf tube and immediately put on ice for transport. Following processing, the samples were transported to and stored in a freezer at −20 °C until enzyme-linked immunosorbent assay analysis (ELISA) was performed. The day ELISA was performed, the samples were taken out of the freezer and thawed at room temperature for 30 min. At that time, the protocol described below was followed to prepare the sandwich ELISA kit (Abcam 190812 Human Tissue Plasminogen Activator SimpleStep ELISA Kit™) for the microplate reader.

tPA analytes were measured using a single-wash (SimpleStep) colorimetric sandwich ELISA assay kit using an Abcam ELISA reader. Each sample was added to well-plate strips pre-coated with immobilization antibodies. A capture and detection antibody cocktail was added to the sample, which was then incubated for one hour. The well was washed, and a detection reagent was added to develop in proportion to the bound complexes. After a 10-min incubation period, a stop solution was added to prepare for quantification. The microplate reader was used to interpret results. The results were recorded in the regulatory binder, with coded values entered into Excel™ and SPSS™ for statistical analysis. The full procedural methods for ELISA analysis are documented in Appendix A (see section on Supplementary materials given at the end of the article).

To refine protocol logistics and reduce the risk of procedural errors, a preliminary analysis was conducted with blood samples from two participants who were not included in the formal study. In addition, this preliminary analysis aimed to determine if the immunoassay was performing as expected and to determine if the investigator was able to achieve results in a reliable manner consistent with those established by experts. Procedures for blood sampling and processing for the preliminary analysis were performed consistent with the methodology described for experimental study participants. The procedural methodology for preliminary analysis is described in Appendix B.

### Statistical analysis

Investigators planned to conduct a mixed-model ANOVA to investigate the differences in serum concentrations of tPA before and after exercise with and without BFR. Before conducting the anticipated mixed-model analysis of variance (ANOVA) between the BFR and work-matched control groups, the assumption of normality was evaluated using Kolmogorov–Smirnov and Shapiro–Wilk tests. The pre-exercise, work-matched control group reached significance, indicating a violation of normality (*P* = 0.000). Corroborating the violation, skewness of the pre-exercise group exceeded 2 (2.76) and kurtosis exceeded 7 (7.71). The assumption for homogeneity of variance was not violated, evident by Levene’s test that failed to reject the null hypothesis (pre-exercise *P* = 0.746; post-exercise *P* = 0.126). Data transformation using both log-transformation and the square root function was unable to normalize the data. As there are no nonparametric test equivalents to the mixed ANOVA, the investigators chose to perform a one-way ANOVA by creating difference scores by taking the post-exercise scores minus the pre-exercise scores between the BFR and work-matched control group. This method allowed the use of bootstrapping to determine confidence intervals when assumptions were not met for the mixed ANOVA.

## Results

Twenty participants were enrolled in the present study. Eighteen participants (mean age: 34.44 ± 9.97) successfully completed the study. One participant was excluded for not adhering to fasting protocol and another due to inability to obtain a post-exercise blood sample. Of the 18 successful participants, nine were male (mean age: 38 ± 11.32) and nine were female (mean age: 30.89 ± 7.39). Table 1 identifies the sex and group allocation of each participant and illustrates the relationship between optical density and tPA concentrations before and after exercise.

**Table 1:** Participant data including sex, group allocation, optical density of sample and tPA concentration values before and after exercise.

Pt no.	Sex	Group	Optical density		tPA concentration (pg/mL)	
			Before exercise	After exercise	Before exercise	After exercise
1	Female	BFRE	0.23	0.28	210.00	282.86
2	Female	BFRE	0.55	0.76	666.43	964.29
3	Female	Control	0.30	0.42	320.00	481.43
4	Male	Control	0.30	0.30	308.57	310.29
5	Male	BFRE	0.30	0.35	316.43	387.89
6	Male	BFRE	0.38	0.50	431.43	592.86
7	Female	Control	0.32	0.31	340.00	329.29
8	Female	BFRE	0.39	0.46	444.29	539.29
9	Male	BFRE	0.41	1.47	480.00	1980.00
10	Female	Control	0.36	0.47	393.57	550.00
11	Male	BFRE	0.53	0.59	653.57	730.31
12	Female	Control	0.36	0.37	394.29	414.29
13	Female	BFRE	0.645	0.7385	806.43	940.00
14	Male	BFRE	0.849	1.204	1097.86	1605.00
15	Male	Control	0.8765	0.88	1137.14	1142.14
16	Male	Control	0.323	0.379	346.43	426.43
17	Male	Control	0.33	0.20	351.43	172.86
18	Female	BFRE	0.54	0.55	656.43	665.71

Pt, participant; tPA, tissue plasminogen activator; BFRE, blood flow restriction exercise.

**Figure 1:**
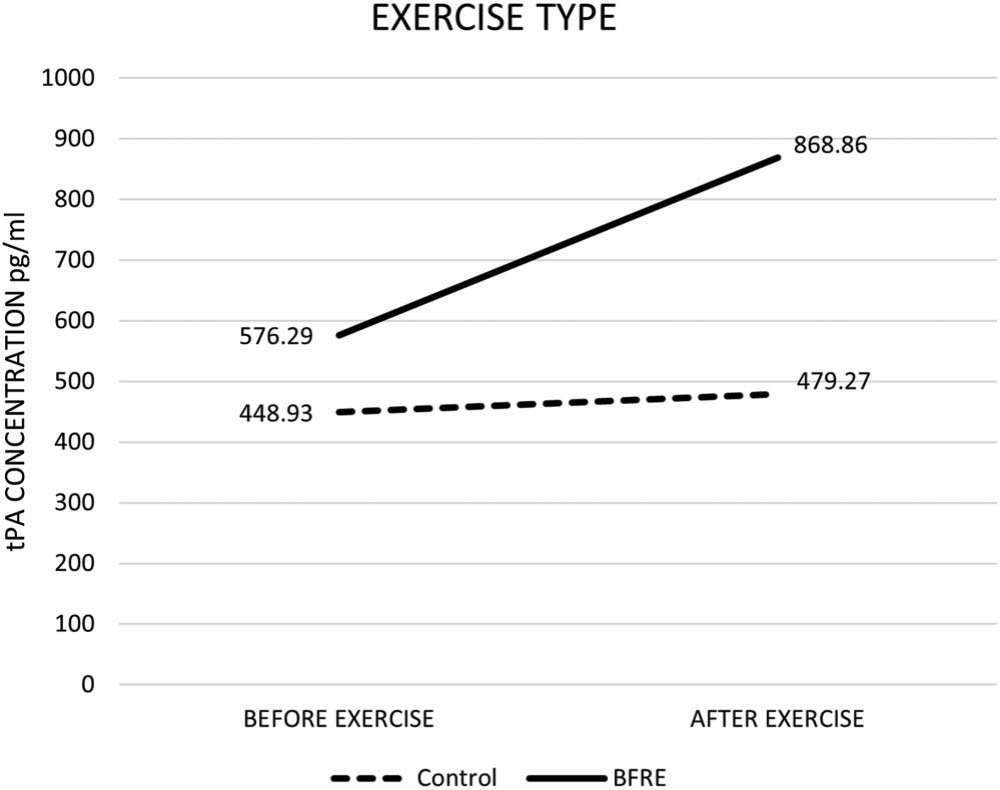
One-way analysis of variance: tPA between groups by exercise type.

Table 2 identifies the descriptive statistics using 95% confidence intervals with bootstrapping. A one-way ANOVA was used to investigate the difference between change scores by exercise type on serum concentration levels of tPA. No significant difference was found between the BFRE and work-matched control group in relation to serum concentrations of tPA change scores (after minus before exercise). However, a large effect size was noted (*F*(1.16) = 2.582, CI = 47.90–375.63, partial eta = 0.139). Figure 1 demonstrates a visual representation of the increased tPA trend in the BFRE group.

**Table 2:** Descriptive statistics with bootstrapping and confidence intervals demonstrating the mean ± SD difference (after minus before) in serum concentrations of tPA between BFRE participants and work-matched controls.

*n*	Exercise group	tPA (pg/mL)*	95% CI
9	BFRE	292.57 ± 448.3683	95.42–599.10
9	Control	30.54 ± 108.2402	−51.07 to 102.26

CI, confidence interval; SD, standard deviation; tPA, tissue plasminogen activator; BFRE, blood flow restriction exercise.

* Values represent mean± SD change pre- to post-exercise.

One-way ANOVA with the between-subjects’ factor (sex) was used to determine the difference between pre- and post-exercise BFRE values of tPA as the dependent variable. Table 3 describes descriptive statistics, including the 95% confidence intervals using bootstrap at 1000.

**Table 3:** Descriptive statistics with bootstrapping and confidence intervals demonstrating the mean ± SD difference in change scores for serum concentrations of tPA before and after exercise between male and female participants using BFR.

*n*	Sex	tPA (pg/mL)*	95% CI
5	Male	248.26 ± 271.22	74.81–1053.81
5	Female	103.97 ± 140.44	39.05–222.98

CI, confidence interval; SD, standard deviation; tPA, tissue plasminogen activator; BFR, blood flow restriction.

* Values represent the mean ± SD change pre- to post-exercise.

**Figure 2:**
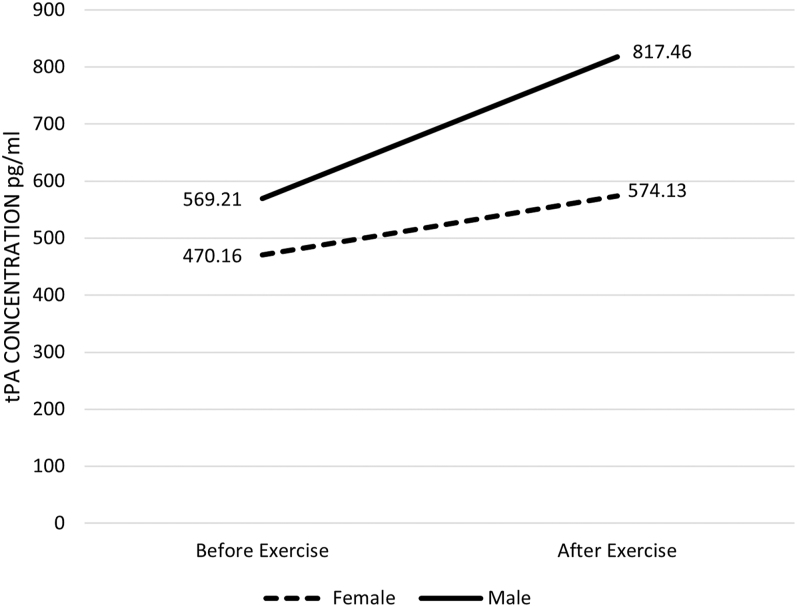
Concentration of tPA before and after exercise, comparing male and female BFRE participants.

Results of the one-way ANOVA found no significant difference between males and females regarding serum levels of tPA change scores on BFRE (*F*(1.8) = 1.539, CI 95.46–610.81, partial eta = 0.161). Fig. 2 provides a visual representation of the changes in the mean levels of serum concentrations of tPA from pre- to post-exercise for males and females using BFR.

## Discussion

The aim of this study was to explore the acute effects of low-load cycling, with BFR applied to one upper and both lower extremities on serum tPA concentration. The authors hypothesized that the participants performing low-load cycling exercise with BFR would have significantly elevated levels of tPA when compared to the work-matched control group without the BFR cuffs. The BFRE group failed to reach significance with tPA compared to the control group and there was no significant difference between male and female groups using BFRE. When exploring the data, the mean concentration of tPA in the BFR group increased by 34% following exercise, whereas the mean concentration of the work-matched control group only experienced a 6% increase. Given the large effect size, investigators speculated that utilizing a larger sample size would have increased the probability of reaching statistical significance. Therefore, a follow-up G power analysis indicated that continuing the methodology until samples from 50 participants were included could likely reach statistical significance.

Two studies, previously described in this manuscript, were able to identify statistically significant increases in tPA concentration with BFRE, utilizing sample sizes similar to the present study. Clark and coworkers found significant increases in serum levels of tPA in a significance in the BFR group (*P* = 0.01) with a similar number of young, healthy participants (15 males, 2 females) performing low-load knee extension exercise with BFR (6). Similarly, Nakajima and coworkers reached significance (*P* = 0.05) in the BFRE group compared to crossover controls in two separate protocols using low-intensity lower extremity exercises (protocol one, *n* = 6; protocol two, *n* = 7) (4).

Several other factors could explain why the present study was unable to reach significance compared to the existing studies. First, the present study used cardiovascular exercise whereas the cited studies used lower extremity exercise strengthening exercises. Second, differences in the type of BFR cuff and occlusion pressures could account for the variation. In addition, Nakajima and coworkers used a hyperbaric chamber in the first protocol and a 24-h bed rest period before exercises in the second protocol (4). Finally, the present study included equal representation between males and females while the previous studies included predominantly male participants.

### Study limitations

The present study examined the effect of BFRE on serum concentration levels of tPA and brain derived neurotrophic factor (BDNF) in healthy adults between the ages of 18–50 years. Therefore, caution should be used with attempts to generalize these results to older adults or those with specific medical diagnoses. In addition, only one bout of BFRE was performed; therefore, it cannot be known from this study if the analytes have a lasting effect on the fibrinolytic and nervous systems.

As with any study using set pressures during BFRE, limitations are a concern. Investigators determined that set pressures would be utilized for the lower and upper extremities based on protocols utilized in similar studies (16, 21). However, due to variations in underlying soft tissue pressure and limb circumference among participants, it is possible that the selected cuff pressures may not have uniformly restricted blood flow across all participants. This potential disparity may have caused a differential effect among participants (24).

The role of hormonal contraceptive use in modulating fibrinolytic activity, particularly tPA, warrants consideration. Prior studies have established that combined oral contraceptives (COCs) influence hemostatic balance, increasing the risk of venous thrombosis while altering levels of coagulation and fibrinolytic factors, including tPA. Women using COCs, especially those containing certain progestins such as desogestrel or drospirenone, exhibit a modified coagulative state, which could confound the observed changes in tPA after BFRE. The meta-analysis by Stegeman and coworkers noted that COCs with specific formulations had a relative risk of venous thrombosis that was significantly higher compared to non-users (25). Since the present study includes both male and female participants, the unequal influence of COCs might have contributed to the variability in tPA response.

### Suggestions for implementation and future research

A prospective, longitudinal, randomized-controlled trial investigating the effects of BFRE compared to standard exercise interventions in persons with active DVT would expand on the current research. In addition to examining potential changes in serum concentrations of tPA, utilizing duplex ultrasound imaging to detect changes in size and overall presence of existing blood clots would broaden the scope of the current research. In addition, a detailed analysis of contraceptive use among female participants and its relationship with tPA concentration could further clarify the findings and improve generalizability.

In recent years, new evidence has suggested that performing low-load exercise coupled with modest blood flow to exercising muscles serves as a potent stimulus for increases in circulating tPA (4, 6). While these studies support lower extremity exercises in homogenous populations, there have been few empirical studies investigating the benefits of cardiovascular exercise on the analyte investigated in the present study.

## Supplementary materials



## Declaration of interest

the authors declare that  there is no conflict of interest that could be perceived as prejudicing the impartiality of the research reported, except as disclosed and cited in the manuscript. 

## Funding

This work did not receive any specific grant from any funding agency in the public, commercial or not-for-profit sector.

## Author contribution statement

JL conceived the study, wrote the paper, performed experiments and analyzed the data. MA conceived the study and edited the paper. IO performed experiments and analyzed the data. LL conceived the study and edited the paper. NA performed experiments. JR analyzed the data and statistics. JF conceived the study and edited the paper. KL performed experiments and analyzed the data.
