# Supplementary material for: Serum tissue plasminogen activator after cycling with blood flow restriction
Source: Vasc Biol. 2025 Feb 17;7(1):e240008. doi: 10.1530/VB-24-0008 (PMC11850040; doi:10.1530/VB-24-0008)
Supplement: Supplementary file 1 [file supplementary_materials.pdf]

APPENDIX A: ELISA ASSAY PROTOCOL

ELISA Analysis Protocol: tPA

Materials

- <u>1X wash buffer:</u> 1X wash buffer is prepared by diluting 10X wash buffer with deionized water. To make 50 mL 1X Wash Buffer PT combine 5 mL Wash Buffer PT 10X with 45 mL deionized water. Mix thoroughly and gently.
- <u>Antibody cocktail:</u> make antibody cocktail by mixing capture and detection antibodies with antibody diluent in the ratio of 1:5. For example, to make 3mL of antibody cocktail, mix equal volume (300uL) of both detectors and capture antibodies with 2.4mL of antibody diluent.
- <u>Standard</u>*:* Always prepare a fresh standard for each experiment. Reconstitute the BDNF standard by adding the volume of sample diluent indicated on the protein vial OR in case volume is not indicated on the vial, add 500uL of sample diluent. This is the stock 20000 pg/mL solution. Add 225uL sample diluent to tube labeled 1 and 150uL to tubes labeled 2-8. From the stock solution, take 75uL of solution and add to tube 1 and from tube 1-8 make a 2-fold dilution leaving tube 8 as blank.

Methods

1. Add 50 µL of all samples or standard to appropriate wells.
2. Add 50 µL of the Antibody Cocktail to each well.
3. Seal the plate and incubate for 1 hour at room temperature on a plate shaker set to 400 Rpm.
4. Wash each well with 3 x 350 µL 1X Wash Buffer PT. Wash by aspirating or decanting from wells then dispensing 350 µL 1X Wash Buffer PT into each well. Wash Buffer PT

should remain in wells for at least 10 seconds. Complete removal of liquid at each step is essential for good performance. After the last wash, invert the plate and tap gently against clean paper towels to remove excess liquid.

5. Add 100 µL of TMB Development Solution to each well and incubate for 10 minutes in the dark on a plate shaker set to 400 rpm. Given variability in laboratory environmental conditions, optimal incubation time may vary between 5 and 20 minutes. The addition of Stop Solution will change the color from blue to yellow and enhance the signal intensity about 3X. To avoid signal saturation, proceed to the next step before the high concentration of the standard reaches a blue color of O.D.600 equal to 1.0.
6. Add 100 µL of Stop Solution to each well. Shake plate on a plate shaker for 1 minute to mix.
7. Record the OD at 450 nm. This is an endpoint reading.
8. Alternative to step 7: Instead of the endpoint reading at 450 nm, record the development of TMB Substrate kinetically. Immediately after addition of TMB Development Solution, begin recording the blue color development with elapsed time in the microplate reader prepared with the following settings: #SampleDilution# 1:4 (TPA)

APPENDIX B: ESTABLISHMENT OF PROCEDURAL RELIABILITY THROUGH PRELIMINARY ANALYSIS

To establish procedural reliability, Abcam, the company from which the ELISA kits were purchased, recommends creating and analyzing duplicates of standard concentration solutions and participant samples to provide adequate data for statistical validation of results. Once the standard concentration duplicates were developed, the average absorbance values for each set of

standard solutions and participant samples could be obtained from the microplate reader and used to calculate analyte concentration in units of picograms per milliliter.

A standard preparation procedure was carried out for tPA. The tPA was reconstituted with normal saline yielding 20,000 pg/ml of stock solution. In the first well of the microplate, 225 microliters of normal saline were added with 150 microliters added to wells two through eight. In the first well, 75 microliters of stock solution were transferred using a micropipette. The first well had a known concentration of tPA at 5,000 pg/ml. The serial dilution continued with transference of 150 microliters of the 5,000 pg/ml from the first well to the second well containing 150 microliters of normal saline yielding 2,500 pg/ml of tPA. Each dilution reduced the tPA concentration by half until there was no tPA remaining in the eighth well. The same procedure was performed to create a standard control duplicate. For analysis of the preliminary sample, a four-fold dilution factor was performed as follows: 150 microliters of normal saline were added to four wells of a microplate. In the first well, 50 microliters of serum sample were added. The first well was diluted by one-fourth (150 microliters of normal saline plus 50 microliters of sample). Fifty microliters of the one-fourth diluted serum sample was transferred into the second, third, and fourth wells, respectively. The sample had only four serial dilutions reducing tPA concentration by four-fold with each transfer. The same procedure was carried out to create a duplicate of the preliminary analysis sample. The sandwich ELISA protocol was performed on the standard controls and preliminary analysis samples. Table 1a shows absorbance quantified in optical density (OD) which was identified by the microplate reader.

Standard Concentration of tPA (pg/ml)	Standard Control Absorb-ance (OD)	Standard Control duplicate Absorb-ance (OD)	Average Standard Absorbance (OD)	20% of mean Standard Duplicates	Abcam Absorb-ance value (OD)	Preliminary Sample Absorbance (OD)
5000	3.114	3.941	3.527	0.7054	3.56	0.282
2500	2.015	1.875	1.945	0.389	2.60	0.28
1250	1.044	0.834	0.939	0.1878	1.38	0.124
625	0.567	0.503	0.535	0.107	0.71	0.136
312.5	0.305	0.273	0.289	0.0578	0.38	0.088
156.25	0.17	0.166	0.168	0.0336	0.21	0.086
78.13	0.108	0.105	0.106	0.0212	0.13	0.063
0	0.082	0.078	0.08	0.016	0.05	0.07

Table 1a: Optical density values for standard control and preliminary sample analysis of tPA

All the standard control and sample duplicates were within 20% of the mean of the duplicates, indicating consistency and reliability of data. The standard means were correlated with the Abcam reference values. The concordance reduces error improving the reliability and internal validity of the experiment. Once optical densities were obtained, standard curves were constructed in immunoassays to interpolate concentrations of the analyte of interest (Figure 1a).

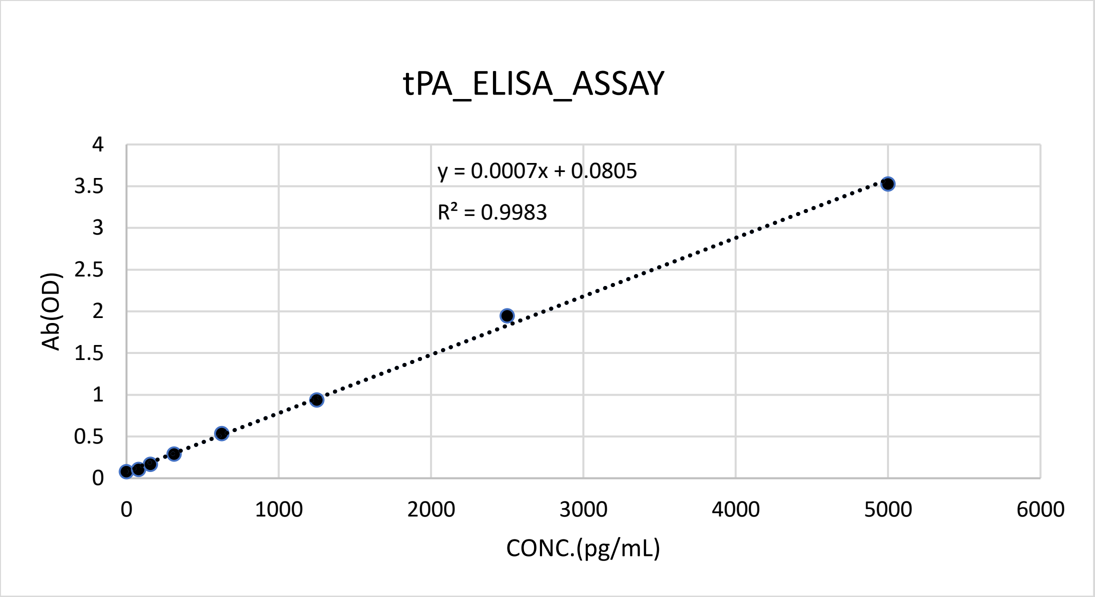

Figure 1a: Standard curve for tPA analyte concentration in preliminary analysis samples

A strong linear relationship was found to exist between optical density and the concentration of tPA in pg/ml (R^2 = 0.9983) revealing reliable data. To convert tPA concentration from optical density to pg/ml, x is solved for in the slope intercept equation (see below).

Samples	OD Average	Con.pg/mL
**1**	**0.281**	**390**
**2**	**0.13**	**71**
**3**	**0.087**	**9**
**4**	**0.0665**	**0**

Table 2a: Sample concentrations of tPA interpolated from standard curve.

Note the mean of the duplicate tPA levels were too low in the most diluted wells to detect any tPA activity. The results of this preliminary data are invaluable for ensuring the correct sensitivity to detect a range of BDNF and tPA values.
